# Interpersonal Determinants of Suicide Risk Among Young Adults: A Cross-Cultural Study

**DOI:** 10.3390/ejihpe16010004

**Published:** 2025-12-24

**Authors:** Noelia Lucía Martínez-Rives, Pilar Martín Chaparro, Yasuhiro Kotera

**Affiliations:** 1 Department of Psychiatry and Social Psychology, University of Murcia, 30100 Murcia, Spain; noelialucia.martinezr@um.es; 2 Faculty of Medicine & Health Sciences, University of Nottingham, Nottingham NG7 2TU, UK

**Keywords:** suicide risk, interpersonal competence, social support, interpersonal theory of suicide, perceived burdensomeness, thwarted belongingness, cross-cultural, Spain, Japan, young adults

## Abstract

(1) Background: Understanding suicide risk across cultures requires examining both universal and culturally specific factors that inform assessment and intervention. This study explores the influence of interpersonal variables—such as interpersonal competence, perceived social support, and constructs from the Interpersonal Theory of Suicide (ITS)—on suicidal behaviour in two culturally distinct samples: young adults from Spain and Japan. (2) Methods: A total of [437] participants (Spanish sample: *n* = 260; Japanese sample: *n* = 177) completed validated measures assessing suicide risk, depression, perceived burdensomeness, thwarted belongingness, acquired capability for suicide, interpersonal competence, and perceived social support. Moderated mediation and SEM comparative analyses were conducted to identify predictors of suicide risk in each cultural context. (3) Results: Social support was a consistent protective factor against depression and suicidal ideation, and interpersonal competence showed more contextual protective effects, significant only in the Japanese sample. Perceived burdensomeness stood out as a robust predictor of depression and suicidal ideation in both samples, and suicidal ideation was strongly associated with suicidal behaviour, while acquired capability for suicide and interpersonal competence did not show a direct association with it. (4) Conclusions: The findings highlight the protective role of interpersonal competence and perceived social support in the progression to suicidal behavior, suggesting cultural similarities and differences in how these factors operate.

## 1. Introduction

### 1.1. Suicidal Behaviour and the Interpersonal Theory of Suicide

Suicide continues to represent a major cause of unnatural death among young people globally (World Health Organization, 2025). Building upon our previous works that provided detailed epidemiological data (Martínez-Rives et al., 2025a), this study extends the analysis to the interpersonal and cultural mechanisms that may explain cross-cultural differences in suicide risk, focusing on the transition from suicidal ideation to suicidal behavior, which is still poorly understood (Klonsky et al., 2021). Specifically, we adopt the ideation-to-action framework, which emphasizes that suicidal thoughts and suicide attempts, although related, undergo distinct processes with different predictors and explanatory mechanisms (Harmer et al., 2025). A key element in this framework is the concept of capability for suicide, understood as the psychological and physiological capacity that allows an individual to progress from ideation to action (Joiner, 2005). The Interpersonal Theory of Suicide (ITS) highlights the role of acquired capability for suicide, manifested through reduced fear of death and increased tolerance for pain as a result of repeated exposure to distressing or painful events, which, added to thwarted belongingness and perceived burdensomeness, facilitates the transition from suicide ideation to behavior (Joiner, 2005).

Within the ideation-to-action framework, the concept of ‘pre-suicidal attempts’, understood as preparatory actions that preceded a suicide attempt, is proposed. Individuals classified as pre-suicidal attempters display higher scores in ‘fearlessness about death’ and suicide risk than individuals only reporting suicidal ideation. By addressing this transitional stage, this framework highlights the need to examine suicide predictors and moderators (Hou et al., 2024). 

### 1.2. Suicide Vulnerability Factors

Some countries have already attempted to screen vulnerable populations for suicide risk factors such as ‘loneliness’. A national survey of 20,000 people about the situation of loneliness and isolation in Japan was conducted in 2024 (Cabinet Office, 2025) to examine which groups were more socially vulnerable and isolated. The age groups between 20–29 and 30–39 years old reached the highest percentages (7.4% and 6%, respectively). Overall, approximately 40% of respondents reported experiencing loneliness to some degree, while 40.6% indicated feeling lonely ‘rarely’ and 18.4% ‘never’. Family bereavement (24.6%), living alone (18.8%) and disturbances in studies and work (14.7%) were the main contributors to loneliness. Regarding interpersonal contact, 9.3% expressed they were not having ‘face-to-face’ talks with family and friends outside the living unit, and 50.6% admitted they were not participating in any social activity.

In Spain, unwanted loneliness or isolation is also mentioned as one of the main risk factors for suicide, considered both at an individual and social level (Madrid + Salud, 2025). The Action Plan for Suicide Prevention 2025–2027 (Comisionado de Salud Mental, 2025), recently implemented in Spain, includes adolescents and young adults’ groups as two of the most vulnerable populations due to an increase in emotional suffering. 

Some studies on predictive factors related to suicide in similarly aged college students reported that depression, between other variables like loneliness or hopelessness, is an important factor in suicidal ideation (Pervin & Ferdowshi, 2016; Byun et al., 2020). More recently, Hou et al. (2024) pointed to depression as a significant predictor of pre-suicidal attempts, alongside the variables contemplated in the ITS: thwarted belongingness, perceived burdensomeness, fearlessness about death (included within the acquired capability for suicide), and suicidal ideation. Furthermore, depression was strongly associated with both suicidal ideation and the preparatory behaviors before attempts. The study of Gregory et al. (2023) talked about how depression not only generates negative emotions but also interferes with the ability to adequately communicate positive emotions. This negatively affects relationships and weakens the protective role of communication and social support.

### 1.3. Interpersonal Competence and Social Support

Previous findings have suggested that proximity or closeness to others without effective communication can mask changes in suicidal behavior, making them less likely to be detected. Consequently, effective communication may be a relevant factor in preventing the transition from ideation to action, because it allows the detection of signs and provides opportunities for timely intervention. Furthermore, broader perceived social support may protect against suicidal ideation by decreasing burdensomeness or thwarted belonging, even before suicidal intent or attempts appear. Consequently, perceived social support could shape the impact of interpersonal communication (Martínez-Rives et al., 2025b).

A systematic review by Gariépy et al. (2016), including Western countries, highlighted that the sources of social support differed across life stages, with spousal support being central during adulthood, followed by family and friends. For example, among the sample of young adults with a history of out-of-home care in the study of Evans et al. (2022), family and material support were found to be associated with greater life satisfaction, while informational and material support were related to fewer mental health symptoms.

Regarding informational support, Cholappallil et al. (2021) analyzed how interpersonal communication skills related to the quality of friendships in emerging adulthood, with indicators of self-confidence, belonging, and well-being. These findings point to a protective role of interpersonal competence by strengthening social integration, a particularly meaningful effect considering that thwarted belongingness is a core risk factor for suicidal behavior within the ITS framework (Joiner, 2005). Referring to communicating tendencies, sex differences have been found. In the study by Hofmann and Wagner (2023), which retrospectively analyzes the behavior and communication skills of men who died by suicide, it was found that they expressed suicidal thoughts mostly indirectly, showing behavioral changes poorly recognized by those around them. These results were reinforced in a recent study by Hansson et al. (2025), where a higher percentage of women (47%) expressed some form of verbal suicidal communication compared to men (23%) and were more likely to contact with health services prior to suicide (72% women vs. 46% men). These sex differences reflected greater expressiveness and verbalization of distress in women, while men may have reported more silent or indirect communication. These findings align with previous research showing a higher percentage of women vulnerable to suicidal thoughts (Jeong-mi & Ji-hoon, 2024). The study by Li et al. (2023) supported the idea that social support helped to protect against depression by reducing loneliness and specific symptoms such as anhedonia, highlighting its variability across all depressive symptoms depending on sex. Mecha et al. (2023) also emphasized the protective role of social support against symptoms of depression and anxiety in young adults, using a Spanish sample, finding that social support, especially from family, was associated with higher levels of resilience.

Receiving social support requires clear and appropriate communication to express emotions and needs. Thus, interpersonal competence mediates access to social support, as it influences the quantity and the quality of social support networks. On the one hand, communication difficulties can lead to social isolation and, therefore, a lower perception of support. On the other hand, an environment that shows empathy encourages openness, and nonjudgmental communication allows people to share their difficulties and seek support when needed. The literature suggests a wide range of interpersonal skills that can contribute especially to suicide detection among young people. As an example of this, closeness with others has been highlighted as a protective factor in life-threatening circumstances, as have the expression of emotions and the perception of emotional and informational support, as they facilitate disclosure and timely help-seeking (Martínez-Rives et al., 2024).

Despite the historical emphasis on psychiatric diagnosis in understanding suicide, the current literature points to a more contextual and functional explanation within a complex network of meanings, social relationships, and life circumstances (Al-Halabí & Fonseca-Pedrero, 2024). From this perspective, it is essential to explore variables such as interpersonal competence or social support as subjective dimensions that mediate the experience of suffering and the search for help.

### 1.4. Prior Evidence and Study Rationale

A recent systematic review and meta-analysis about clinical predictors of suicidal behavior in depression showed characteristic symptoms of this disorder as significant predictors of suicide, such as severity, suicidal ideation, and hopelessness (Riera-Serra et al., 2024). Our previous study (Martínez-Rives et al., 2025d) represented an initial exploration of the core variables of the ITS of Joiner (2005) with the same sample of young adults from Spain and Japan using simpler analysis. Through regression models, we could analyze the relationship between its key variables (perceived burdensomeness, thwarted belongingness, and acquired capability for suicide) and suicidal risk and depressive symptoms, revealing relevant cultural differences. Perceived burdensomeness was significantly higher in the Japanese sample and had greater relevance in both cultures. Acquired capability showed greater impact in Spain, whereas thwarted belongingness had more influence in Japan. However, that analysis did not test the full theoretical model of ITS, as the moderating role of capability in the transition from suicidal ideation to behavior was not examined. The results were interpreted cautiously, avoiding more complex associations between variables. The present work advances this line of research by proposing a more complex analysis tailored to Joiner’s original proposal and incorporating protective factors such as ‘interpersonal competence’ and ‘perceived social support’. In this way, the aim is not only to expand previous findings but also to reassess acquired capability for suicide as a moderating variable offering empirical evidence into factors associated with the transition from suicidal ideation to behavior.

Models like the ITS underscore the need to examine not only risk factors for suicidal ideation but also the mechanisms and protective variables that can influence whether an individual progresses to suicidal behavior (Harmer et al., 2025). These differences lay the groundwork for this research on how interpersonal factors relate to suicidal risk in different cultural contexts, Spain and Japan in this case. Japan is not as collectivist as other Asian societies (China, Korea), and in terms of individualism, it is quite similar to Spain (62 vs 67). However, the cultural ways of relating and communicating are different: they are more open and relational in Spain and situational and reserved in Japan (The Culture Factor Group, n.d.). In a study by Shen et al. (2024), a collectivist dimension and high-context communication, together with hierarchical structures, was used to explain how social norms, cultural values, and interpersonal dynamics influence ways of communicating.

In conclusion, the present research expands the previous analysis by incorporating communication skills and perceived social support as potential protective factors within the ITS framework. This approach aims to examine how interpersonal and social resources may moderate or buffer the effects of associations between ITS variables on suicide risk. Additionally, we propose exploring depression as a dependent variable, assessing possible cultural differences in these effects.

### 1.5. Objectives, Hypotheses and Research Questions

This study aims to analyze the mediating role of interpersonal competence and perceived social support in variables apart from the ITS, examining their effects on suicide risk, and in depression, as a comparative outcome variable, in young adults from two different cultural origins: Spain and Japan. Through this comparative and intercultural approach, the study aims to provide a more nuanced understanding of suicide risk in different cultural contexts.

Hypotheses:

**H1:**  *Interpersonal competence and perceived social support are negatively associated with ‘depression’ and ‘suicidal risk’. These associations are expected to be stronger in the Spanish than in Japanese, where communication tends to be more indirect and social support more implicit*.

**H2:**  *‘Perceived social support’ moderates the association between thwarted belongingness and/or perceived burdensomeness on ‘suicidal ideation’, and ‘interpersonal competence’ moderates the transition from ideation to behavior, in which the acquired capability for suicide interferes. Therefore, the negative impact of ‘thwarted belongingness’ or ‘perceived burdensomeness’ on ‘suicidal ideation’ is weaker in people with better perception of social support, and the impact of ‘acquired capability for suicide’ is less significant on ‘suicidal behavior’ in people who are more competent at an interpersonal level*.

**H3:**  *The relationship between perceived burdensomeness, thwarted belongingness, and ‘suicidal ideation’ is stronger in the Japanese than in the Spanish, reflecting cultural differences in the emotional expression and in the concept of ‘being a burden’ or ‘not belonging’*.

**H4:**  *The acquired capability for suicide moderates the transition from ideation to behavior. This association is expected to be stronger in Spain, given previously reported differences in fearlessness about death*.

*Based on these hypotheses, the following research questions are proposed:*

- *Are there differences in the associative weight of thwarted belongingness and perceived burdensomeness on suicidal ideation, when adjusted for social support?*
- *Does interpersonal competence moderate the relationship between acquired capability for suicide and the transition from suicidal ideation to behavior?*
- *Are the associations of social support and interpersonal competence through the ITS variables different in each cultural context (Spain vs. Japan)?*
- *Are the same interpersonal and cultural factors associated with suicide risk also associated with depressive symptoms?*

## 2. Materials and Methods

### 2.1. Study Design and Setting

This study follows an observational, cross-sectional and correlational design. 

### 2.2. Inclusion and Exclusion Criteria

The inclusion criteria were being a young adult between 18 and 40 years old of Spanish or Japanese nationality. Participants of Spanish or Japanese descent born and residing in countries different from the country of origin were excluded.

### 2.3. Participants and Procedures

The sample used in this study corresponds to that collected within the framework of a doctoral project approved by the Ethics Committee of the University of Murcia (ID: 4080/2022). Briefly, it consisted of 437 young adults between the ages of 18 and 40, with 260 participants from Spain (51.5% women, M = 31.08, SD = 5.54) and 177 from Japan (52% women, M = 29.08, SD = 5.65). In the Spanish sample, most participants were single (73.1%), with university-level education (around 66%); most of them lived with someone else (45% lived with their partners and children and 23.8% with their parents and/or siblings); and the majority were employed (approximately 64%). In the Japanese sample, most participants were also single (63.8%), with higher education (over 70%); most of them 39.5% lived alone; 23.7% lived only with their partner; and 45% were employed full-time. The percentages of the new sociodemographic variables incorporated in this study, based on the total sample, can be found in Table 1.

Recruitment was carried out through two channels: through academic contacts using a snowball strategy and through the Prolific platform, with the offer of financial compensation. Participation was voluntary and anonymous, after informed consent was obtained online. In this study, we added more sociodemographic variables to those already collected (age, sex, occupation…), such as mental and physical health diagnosis, received treatment, economical status, and areas of life affected by COVID-19.

### 2.4. Variables and Measures

In the present study, we used the same sample and core measures as in our previous exploratory work; these were the Interpersonal Needs Questionnaire (INQ; Van Orden et al., 2012) to assess thwarted belongingness and perceived burdensomeness; the Acquired Capability for Suicide Scale-Fearlessness About Death (ACSS-FAD; Ribeiro et al., 2014); the Patient Health Questionnaire-9 (PHQ-9; Kroenke et al., 2001) to assess depressive symptoms; and the Columbia-Suicide Severity Rating Scale, Screen Version (C-SSRS; Posner et al., 2011) to assess suicide risk, all in their Spanish and Japanese versions. In the current study, the acquired capability for suicide was considered as a moderating variable in the transition from ideation to suicidal behavior. Thus, the PHQ-9 and C-SSRS (ideation and behavior) were defined as dependent variables; the INQ-TB (thwarted belongingness), INQ-PB (perceived burdensomeness), and ACSS-FAD were included as predictors; and the Interpersonal Competence Questionnaire (ICQ-15; Coroiu et al., 2015) and Multidimensional Scale of Perceived Social Support (MSPSS, Zimet et al., 1988) were incorporated as potential protective mediators.

Detailed descriptions of these instruments, including their psychometric properties, in their Spanish and Japanese versions were already reported in our previous exploratory study. Consequently, they are not reiterated in this manuscript, although all measures used correspond to validated versions in both languages (e.g., Aiba et al., 2019; Fonseca-Pedrero et al., 2023).

C-SSRS screen version (Posner et al., 2011).

Since the screen version of the C-SSRS does not have published psychometric properties, the present study evaluates its reliability and validity in the Spanish and Japanese samples. Internal consistency indices have been calculated in both samples, and correlations between items and factors have been examined to ensure the measure’s suitability for subsequent analyses. Although its clinical structure groups the items differently (1–2; 3 and 6.1; 4–5 and 6.2), for this research, it was necessary to divide them according to the model that differentiates ideation from behavior. For instance, items measuring suicidal ideation included questions such as ‘Have you wished you were dead or wished you could go to sleep and not wake up?’, whereas items measuring suicidal behavior included ‘Have you ever done anything, started to do anything, or prepared to do anything to end your life?’. According to this, items 1–5 were grouped for the ‘ideation’ scale and 6.1–6.2 for ‘behavior’. This structure was analyzed to ensure its internal consistency.

The measure of ‘suicidal ideation’ was ordinal, taking 0 (out of risk) if the answer was ‘no’ to items 1–5; 1 (low risk) of ideation if the answer was ‘yes’ only to items 1, 2 or both; 2 (medium risk) if the answer was also ‘yes’ to item 3; and finally 3 (high risk) if the answer was ‘yes’ to items 4, 5 or both. The measure of ‘suicidal behavior’ was binary, being 0 if the answer to 6.1, 6.2, or both was ‘no’ and 1 if the answer to 6.1, 6.2. or both was ‘yes’.

As for the two protective mediator measures included in this article, the following description is included:

Multidimensional Scale of Perceived Social Support (Zimet et al., 1988).

The MSPSS consists of 12 items. These are divided into three factor groups with four items each depending on the source of support (family, friends, or significant other). Example items include ‘My family really tries to help me’ (family), ‘I can talk about my problems with my friends’ (friends), and ‘There is a special person who is around when I am in need’ (significant other). Its Likert rating scale has 7 points, from very strongly disagree (1) to very strongly agree (7). Regarding its psychometric properties, the Cronbach’s coefficient alpha values for the Significant Other, Family, and Friends subscales were 0.91, 0.87, and 0.85, respectively, and the value for the total scale was 0.88, showing good internal reliability. It also demonstrated stability over time, as the test–retest coefficients for the Significant Other, Family, and Friends subscales were 0.72, 0.85, and 0.75, respectively, and 0.85 for the whole scale. Finally, its construct validity was evident as the MSPSS subscales were significantly inversely related to depression and anxiety subscales of the Hopkins Symptom Checklist (Zimet et al., 1988).

Their Spanish (Landeta & Calvete, 2002) and Japanese (Iwasa et al., 2007) versions also showed good psychometric properties. The Spanish version’s Cronbach’s coefficient alpha values were very similar to the original in the different subscales and 0.89 in the total scale. The homogeneity indices were at least 0.42 or higher. In addition to this, the correlation coefficient values with scales that measure different concepts, such as hostility, were negative or low (Landeta & Calvete, 2002). The Japanese version also showed a three-factor structure like the original version. Cronbach’s alpha values were even higher in three subscales (0.91, 0.94, 0.88, and 0.90, respectively), as an indicator of its high internal consistency. It also demonstrated its criterion validity using the Mental Health Questionnaire (Iwasa et al., 2007).

After checking the distribution of the MSPSS of the total sample, with a mean of 4.13 and median of 5.50, we found a centered distribution on medium-high values (25th percentile = 4.21, 75th percentile = 6.41) with a SD = 1.51. For this purpose, the scale response descriptors were used as a guide for the participant results. Therefore, scores between 1 to 2.9 were considered low support; a score of 3 to 5 was considered moderate support; and a score from 5.1 to 7 high support.

Interpersonal Competence Questionnaire-15 (Coroiu et al., 2015).

The ICQ-15 is a brief form of the 40-item questionnaire by Buhrmester et al. (1988) with a 5-point Likert-type response scale, focused on five areas of interpersonal competence: (1) initiating relationships and/or conversations—‘Introducing yourself to someone you might like to get to know/date’; (2) negative assertion—‘Confronting your close companion when he/she has broken a promise’; (3) emotional support—‘Helping a close companion get to the heart of the problem he/she is experiencing’; (4) self-disclosure—‘Letting a new companion get to know the real you’; and (5) conflict management—‘Being able to take a companion’s perspective in a fight and really understand his or her point of view’. 

Its Cronbach’s alpha was 0.83, showing good reliability, with an inter-scale correlation value of 0.55 and higher, and its inter-item correlations were at least 0.35 or higher (Coroiu et al., 2015).

The Spanish version of the ICQ-15 by Salavera and Usán (2018) showed good construct validity through the exploratory factor analysis (KMO = 0.836; Bartlett *p* < 0.001), explaining 64.88% of the total variance. Pearson correlation values between factors also showed good internal consistency (0.336 and higher with a *p* < 0.1). Regarding its reliability, Cronbach’s alpha yielded a value of 0.85, even higher than that of the original version. Furthermore, the confirmatory analysis demonstrated adequate fit indices. The Japanese version of this instrument was translated and validated by the authors of this article, so its psychometric properties can be consulted in the following study: (Martínez-Rives et al., 2025c). This validation included psychometric analyses such as factor structure, internal consistency, and convergent validity.

More sociodemographic variables added to those already asked (age, sex, occupation, educational level, country of residence, cohabitation unit and years of residence there and marital status) were also included, like mental and physical health diagnosis, treatment received, economic level, and areas of life affected by COVID-19.

### 2.5. Data Analysis

All data analyses were conducted in R program version 4.5.1 (13 June 2025 ucrt), using the package lavaan 0.6–20 for structural equation modelling (SEM). The analyses were made based on a joint dataset and subsequently stratified by country. Descriptive analyses were computed before conducting regression analyses including the moderators (social support and interpersonal competence) and the dependent variable ‘depression’, checking assumptions of normality, homoscedasticity, multicollinearity, and independence of errors. We used residual plots, variance inflation factors (VIF), and the Durbin–Watson statistic to ensure the validity of the results. Using the ‘suicide risk’ as dependent variable, independence of observations, proportionality of odds, and the monotonic relationship between continuous moderators were checked with scatter plots.

Simple correlations were made with the moderators MSPSS and ICQ-15 to analyze which area of social support (friends, family or significant others) and which dimensions of interpersonal competence (negative assertion…) could have more weight over the dependent variables (‘depression’ and ‘suicide risk’). Descriptive statistics and correlations among PHQ-9, C-SSRS, INQ-PB, INQ-TB, and ACSS-FAD were reported in a previous study (Martínez-Rives et al., 2025d). To avoid unnecessary duplication, in the present study, we focused on the additional variables ICQ-15 and MSPSS and their associations with the ITS variables and depressive symptoms.

In the present study, we focused on the inclusion of additional variables, namely communication skills (ICQ-15) and perceived social support (MSPSS), to examine their associations with the ITS-related variables (INQ-15 and ACSS-FAD) and depressive symptoms (PHQ-9). Descriptive statistics and correlations for ICQ-15 and MSPSS and their relations with the previously studied variables are reported here to provide a complete overview of the current sample.”

For the PHQ-9, depression was treated as a continuous variable, so multiple linear regression analyses were conducted, as SEM was not required to model phase transitions (unlike suicidal ideation and behavior). Given the ordinal nature of the C-SSRS screening items, ‘suicidal ideation’ (items 1–5) was analyzed using ordinal regression models, which are more appropriate for ordered categorical outcomes. As an ordinal model for ‘suicide ideation’, the significance of the coefficients was interpreted based on the t values (|t| ≈ 2 equivalent to *p* ≈ 0.05).

After an ordinal regression model statistically predicting ‘suicidal ideation’, Structural Equation Modelling (SEM) was used to follow the theoretical model of the Interpersonal Theory of Suicide and to add the moderation of interpersonal competence through the ICQ-15 in the transition to ‘suicidal behavior’ (binary). In the regression models of the SEM, we checked std.all (fully standardized coefficient) values, as we made comparisons between variables and between groups (Spain vs. Japan). Finally, logistic regression analysis was conducted for a more direct examination of the transition from suicidal ideation to behavior, including the previous dependent variable ‘suicidal ideation’, now entered as a binary variable and predictor of suicidal behavior. In this step, the independent variables INQ-PB and INQ-TB were embedded within the suicidal ideation variable.

### 2.6. Measurement Model

The measurement model specified latent constructs according to the Interpersonal Theory of Suicide. Paths were defined from INQ-TB and INQ-PB to suicidal ideation, and from suicidal ideation adding ACSS-FAD to the transition to suicidal behavior. MSPSS and ICQ-15 were incorporated as moderators into the structural model. To avoid overparameterization and preserve model parsimony of the SEM model for C-SSRS with many fixed covariates, we only included ‘country’ and ‘sex’, focusing on modelling the theoretical relationships between the main latent constructs. Although some of the sociodemographic variables may influence suicidal ideation and behavior, their simultaneous inclusion increased the risk of collinearity and reduced the degrees of freedom, compromising the stability of the estimates. Consistent with methodological recommendations, variables of theorical interest were prioritized, while broader sociodemographic factors (i.e., marital status or occupation) were reported as context rather than as key explanatory factors (López-Roldán & Fachelli, 2015).

Model estimation used WLSMV using the lavaan package (version 0.6–20) for ordinal indicators (C-SSRS). Standardized coefficients (std.all) were examined for comparisons across variables and groups (Spain vs. Japan). Full Confirmatory Factor Analysis (CFA) loadings and alternative model comparisons (three-factor structure vs. two-factor structure) are provided in the Supplementary Materials S1. To compare the results with the other dependent variable ‘depression’, the process is divided into two stages.

Stage 1: Examination of the associations between ‘thwarted belongingness’ and ‘perceived burdensomeness’ with ‘depression’ and testing the moderation of ‘social support’.

Stage 2: Examination of the associations between ‘acquired capability for suicide’ and ‘depression’ when added to the previous variables and testing the moderation of ‘interpersonal competence’.

## 3. Results

### 3.1. Perceived Social Support and Interpersonal Competence as Protective Moderators

Descriptive statistics for social support and interpersonal competence (assessed using the MSPSS and ICQ-15, respectively) can be found in Supplementary Material S2, and their direct effects on suicide risk and depression can be found in Supplementary Material S3. Before proceeding with the complex analyses, we centered the independent variables and moderators scores and checked the basic principles to ensure the validity of the results.

In the first place, a multiple linear regression analysis was performed to explore the effect of perceived social support (MSPSS) and interpersonal competence (ICQ-15) on depression, as measured by the PHQ-9. Levene’s test for homogeneity of variances found F = 0.019 df1 = 1 df2 = 435 Sig. = 0.890 (>0.05), indicating that the homoscedasticity assumption was met. The results of inter-subject effects tests for PHQ-9 on the overall model explained 5% of the variance (adjusted model F = 7.646, *p* < 0.001). For this reason, although the total effect was small, the overall model was significant. Through the individual effects of MPSS and ICQ-15, only MPSS significantly affected the results of PHQ-9 (F = 12.864, *p* < 0.001, Partial η^2^ = 0.029, Power = 0.947). Even though the model was significant, the explained variance was low, so other factors probably had an influence on the PHQ-9. The modified Breusch–Pagan test found a Chi-square = 0.589 (df = 1, Sig. = 0.443 > 0.05), so there was no evidence of heteroscedasticity. Checking the observed and predicted plot in Figure S1 (see Supplementary Material S4), no curvilinear pattern is noticeable; the residual deviance plot looks homogeneous; and reasonably random dispersion is observed, without a strong trend. Thus, the linearity assumption is met acceptably. Moreover, the tolerance is over 0.2 (0.766), and the VIF > 5 (1.306), showing no multicollinearity problems.

The Durbin–Watson statistic is 1.519 (close to 2), indicating independence of errors. The histogram and P–P plot of the residuals show a reasonable fit to normality, with no serious deviations.

Using the results of the C-SSRS as a dependent variable with the moderators MSPSS and ICQ-15, we can prove the independence of the observations, as each participant is an independent case from an individual questionnaire. Through an ordinal regression analysis with ‘social support’ and ‘interpersonal competence’ as predictors, the log likelihood (–2LL) decreases from 1062.551 to 1047.545 and Chi-square = 15.006 (df = 2; *p* = 0.001), so we conclude that these variables are significantly associated with suicide risk. The Cox and Snell values (0.034) and Nagelkerke (0.037) indicate small variance explained in suicide risk (3–4%). As with the model with the PHQ-9, ‘social support’ turned out to be a significant protective factor with an estimation of −0.241 (*p* < 0.001). Finally, we confirmed the validation of the ordinal model through the Test of Parallel Lines (*p* = 0.291, >0.05), concluding that the coefficients were the same at all levels of suicide risk.

In both models, using either the PHQ-9 or the C-SSRS, only the MSPSS showed negative and significant coefficient scores (−0.792, *p* < 0.01; and −0.241, *p* < 0.01, respectively). For the ICQ-15, the coefficient was negative but not significant in the PHQ-9 model (−0.170, *p* = 0.690) and was even positive in the C-SSRS model (0.78, *p* = 0.553). When including the fixed ‘country’ factor, no significant differences were found in the PHQ-9 scores (F = 3.047, *p* = 0.082), whereas significant differences emerged in the C-SSRS scores, with a coefficient value of −0.543 (*p* = 0.003) for the Spanish sample.

#### 3.1.1. Moderation of Perceived Social Support and Interpersonal Competence in Depression

Since the score on the PHQ-9 is a continuous variable that was not divided into dimensions, like the C-SSRS screen version in ideation and behavior, we stuck to the results of the stepwise regression analysis that we previously performed in the first exploratory analyses. As a result, we found that both in the Japanese and Spanish samples, model 1 included INQ-PB (Beta = 0.97 and 0.62, respectively, *p* < 0.001), but model 2 added ACSS-FAD in the Spanish sample (Beta = 0.036, *p* = 0.017), and INQ-TB was added in the Japanese version (beta = 0.184, *p* < 0.05).

Using a hierarchical linear regression model on the PHQ-9 with the variables INQ-PB and INQ-TB with the moderator MSPSS and covariates (country and sex), we find the following results, as shown in Table 2:

As we can see from the coefficient values, although both INQ-PB and INQTB positively correlate with the results on PHQ-9, only INQ-PB does so significantly (*p* < 0.001). Considerable differences can also be found by country (−4.48, *p* < 0.001); as already pointed out, Japan had lower scores on PHQ-9. In the general sample, sex differences were pointed out, as men had a score of −0.93 (*p* < 0.01). MSPSS correlated negatively with PHQ-9 scores, even though it was not significant. The four-way interaction (INQPB–INQTB–MSPSS–country) coefficient was 0.0015839, showing little difference in this combination between countries, and the difference was not significant. The triple interaction INQ-PB–INQ-TB–MSPSS was not significant but was less so than the double interaction INQ-PB–INQ-TB. The main influence of INQ-PB was higher in the Japanese sample (*p* < 0.001). The mediation of MSPSS, although it was not significant with any of the other independent variables in isolation, was positively significant with INQ-TB in the Japanese sample (*p* < 0.001), as we can see in Figure S2 and the Supplementary Materials S5.

After obtaining slopes of INQ-TB across the different levels of MSPSS (according to the defined scoring ranges) on the PHQ-9 results, the slope remained virtually stable in the Spanish sample across all levels (≈0.02). Nonetheless, in the Japanese sample, the slope was close to zero and non-significant at a low MSPSS level (−0.88), increasing to 0.10 at a medium level (0.37; 95% CI: 0.02–0.18, significant) and reaching 0.18 at high levels (1.29; 95% CI: 0.09–0.27, highly significant).

The three-way and two-way interactions between variables and their significance level can be seen in the following Table 3 and Table 4.

Although the interactions of MSPSS with INQ-PB and INQ-TB were not significant, they were negative. However, the MSPSS did not positively and significantly moderate the interaction between INQ-TB/PB on PHQ-9 in this model. The R^2^ of the overall models was 0.7247 (adjusted = 0.7135); thus, we proved that the model explained the high variance in PHQ-9 results, and F = 64.87 (*p* < 0.001) indicated high significance.

Including ACSS as moderator of the gravity of the depression level, we ran a multiple moderation analysis, adding ICQ-15 as a second-level moderator (see Table 5).

Examining R^2^ = 0.7332 (adjusted = 0.712), we can see that the model explains a high percentage of the variance in the PHQ-9, even when the variable ACSS is not included as a predictor. Focusing on this new variable individually, its coefficient is not significant, although it is positive with PHQ-9 results. The ICQ-15 coefficient with PHQ-9 is positive but not significant. The two-way interactions show a slight decrease in the effect of INQ-TB and ACSS on PHQ-9 with the addition of the ICQ-15 but not of INQ-PB, as well as a tendency to increase the results with any of the combinations of the ITS variables; however, this is not significant.

Through the three-way interaction, we found a negative coefficient between the three variables included in the ITS (INQ-PB, INQ-TB and ACSS), coef = −8.432 × 10^−5^, *p* < 0.88515 with PHQ-9 results. This was also the case for INQ-PB, INQ-TB, INQ-PB, and ACSS with ICQ-15, with the exception of the combination of INQ-TB, ACSS, and ICQ-15, which showed a positive coefficient. Adding the ICQ-15 to the ITS variables, the correlation was negative (−1.887 × 10^−4^) but not significant.

Focusing on country interactions, the Japanese sample in the combination between INQ-TB with ICQ-15 had a positive and significant coefficient (*p* < 0.05), higher than in the Spanish sample. A statistical trend was observed (*p* < 0.1) between INQ-PB with ACSS when adding ICQ-15 to the Japanese sample (2.589 × 10^−2^), and this can be seen in Figure S3 in Supplementary Material S6. In the four-way interaction, adding ACSS to INQ-PB and INQ-TB, the Japanese sample scored 1.987 × 10^−3^ (*p* < 0.01) more than Spanish sample, as we can see graphically in Figure S4 and in Supplementary Material S6. Although we can observe a negative coefficient in the interaction between the ITS variables (INQPB, INQTB and ACSS) with ICQ and between countries, this value was not significant (−1.584 × 10^−4^, *p* = 0.84332).

#### 3.1.2. Moderation of Perceived Social Support and Interpersonal Competence in Suicide

##### Moderation of Perceived Social Support on Suicidal Ideation

To examine how perceived social support influences suicidal risk, first, an ordinal regression model was conducted with suicidal ideation (C-SSRS) as the dependent variable with the predictors ‘perceived burdensomeness’ (INQ-PB) and ‘thwarted belongingness’ (INQ-TB), while ‘perceived social support’ (MSPSS) was tested as a potential moderating factor. As covariates, sex and country were also included. The main results are shown in Table 6, with 95% confidence intervals (CI) for each predictor and their interactions, to identify which predictors have a significant effect on suicidal ideation (C-SSRS) and how that effect varies by country. Only those values where the CI does not include 0 were considered significant:

The model estimated three thresholds for suicidal ideation between C-SSRS categories (−1.12 (0|1), 0.82 (1|2), and 1.62 (2|3)), all statistically significant (*p* < 0.01), confirming the ordinal structure of the dependent variable. In this ordinal model, INQ-PB and INQTB stood out as relevant predictors and MSPSS as protective moderator (coef = −0.24), although in the Japanese sample, the relation was significantly lower in these predictors than in the Spanish sample. The combination of INQ-PB and INQ-TB with MSPSS was positive but not significant in any of the countries. In the Japanese sample, INQ-TB had a negative marginal tendency (coef. = −0.029, t = −1.85) only with MSPSS, which was nearly significant considering the values of the IC [−0.06, 0.00]. Studying the confidence intervals (IC 95%), we can confirm that the main positive and significant effects of the variables were confirmed. Both the interaction between INQ-PB and INQ-TB with MSPSS and the extended model including country as an additional moderator (four-way interaction) yielded confidence intervals that crossed zero ([−0.001243609, 0.005214636] and [−0.004444058, 0.002798033] respectively), indicating non-significant effects.

##### Moderation of Interpersonal Competence on Suicidal Behavior

To analyze the combined effect of INQ-PB and INQ-TB on suicidal ideation and understand its effects on the transition to suicidal behavior, an interaction between these two variables was created (‘predicted ideation’), representing suicidal ideation estimated by the combination effect of INQ-PB and INQ-TB. Suicidal behavior was a binary variable (0/1), so to fit SEM with the WLSMV estimator, it was modelled as an underlying latent ordinal variable, which allowed the estimation of a threshold, separating categories 0 and 1, fixing the variance of the latent variable to 1 according to the convention for dichotomous variables. Then, an SEM model was evaluated including another predictor (ACSS) and the moderator ICQ-15. The results (see Table 7) showed that in this new model, no significant effects were found for ‘predicted ideation’, acquired capability for suicide, interpersonal competence, or their interactions on suicidal behavior. Due to model saturation, sociodemographic variables were not included in this phase of the analysis.

Among all the coefficients, only ACSS as a predictor of suicidal behavior showed a marginal effect. Although it was not significant, it could indicate a trend. Any one of the interactions could be significant in explaining the transition from suicidal ideation to behavior. This could be the result of a saturated model (df = 0), despite CFI and TLI = 1 and RMSEA and SRMR = 0, which indicate a perfect fit.

Since the initial SEM model was saturated and included ‘suicidal ideation’ as a result of the interaction between INQ-PB and INQ-TB, a more parsimonious model was estimated through logistic regression. In this simplified model, the previous dependent variable ‘suicidal ideation’ was included as a predictor, as our preliminary analyses showed that the interaction of INQ-PB and INQ-TB was not statistically significant, thereby reducing the number of estimated parameters, increasing the model’s degrees of freedom, and improving the fit of the model (null deviance:330.75 on df = 436; residual deviance: 266.86 on df = 428; AIC: 284.86). Furthermore, the covariates ‘sex’ and ‘country’ were incorporated into this model in order to control potential sociodemographic differences. To avoid processing problems in the logistic regression, suicidal ideation, now as predictor, was converted into a binary variable, since the main aim was to examine the transition from ideation to behavior, making the level of ideation severity irrelevant in this step. In Table 8, the main results can be found.

The OR values of suicidal ideation showed a robust association with suicidal behavior (~14 times more likely to engage in behavior than those without) but not for ACSS. Although the interactions did go in the desired direction, being positive with ACSS and negative with ICQ-15, they were not significant either. Regarding the covariates, only the factor ‘country’ was significant. The Japanese were more likely to engage in behavior than the Spanish (≈3.5).

## 4. Discussion

### 4.1. Main Findings and Interpretation

The current study examined the cross-cultural relevance of the variables included in the ITS in two different samples, one from the East (Japan) and another from the West (Spain), with the particularities that this entails. Both samples were broadly comparable in terms of key sociodemographic characteristics such as sex distribution, educational level, and employment status. The age range was restricted to young adults (18–40 years), minimizing variability associated with developmental stages. Minor differences emerged regarding living arrangements and relationship status, which are consistent with cultural and social differences between both contexts rather than with sampling bias. Therefore, these variations were unlikely to have significantly affected the observed relationships between the main variables.

In general, the main objective, which was to analyze the mediating role of interpersonal competence and perceived social support on the variables pointed out by the ITS (by examining their effects on suicide risk and comparing with depression outcomes in two different samples), was achieved, although the model did not confirm ‘full mediation’. The existing literature (Caban et al., 2022) has already highlighted that interpersonal communication and social support are central components in mental health interventions among young people, although poorly theorized and operationalized. This reinforces the need to examine not only whether social support and communication are associated with suicide risk but also how their role as mediators within different models can explain it.

At first, simple Pearson and Spearman correlations in the Spanish sample between ICQ-15 and MSPSS with PHQ-9 and C-SSRS results showed that neither social support nor interpersonal competence had a significant direct effect on depression symptoms. Only the area of ‘social support’ showed a relationship with suicide risk, more specifically ‘significant others’. The results of a study with the Spanish population (Simó-Noguera et al., 2015) showed a significant decline in psychological well-being associated with relationship breakdown, with higher levels of depression in both sexes. This aligns with the crucial role of significant others, even more so than family or friends; the presence or absence of a close, intimate, and emotionally relevant figure constitutes a key protective factor.

In the Japanese sample, simple Pearson correlations between ICQ-15 with PHQ-9 results showed a significant correlation with the ‘disclosure’ dimension (r = −0.155, *p* = 0.039). Studies about disclosure in the Japanese population like that by Oishi (2023) about positive expression through writing suggest that promoting safer, less threatening forms of self-expression may buffer the emotional impact of interpersonal difficulties and contribute to better mental health. Uchida et al. (2020) also highlight the benefits of self-disclosure for emotional regulation and social connectedness, while also noting that contextual factors such as gender norms can influence individuals’ comfort with disclosing personal problems. Regarding Spearman correlations between ICQ-15 with C-SSRS, they revealed different important dimensions, as significant negative correlations were found with ‘initiating relationships’ (ρ = −0.206, *p* = 0.006) and ‘conflict management’ (ρ = −0.198, *p* = 0.008). Spearman correlations were also analyzed between MSPSS areas and C-SSRS, where only the negative correlation with ‘family’ support (ρ = −0.289, *p* < 0.01) turned out to be significant. These findings are consistent with previous studies indicating that individuals with suicidal ideation report receiving significantly less family support and feeling less satisfied with that support (Endo et al., 2014). A more recent study (Iwasawa et al., 2025) examined the associations between loneliness, suicidal ideation, and psychological distress, and this relationship persisted regardless of family composition. Although the family constituted the primary source of emotional connection, the underlying mechanism was likely more related to the subjective experience of support rather than to the family structure itself. Different results were found between the MSPSS with PHQ-9, with ‘family’ support being the most associated (−0.408, *p* < 0.001), followed by ‘significant other’ support (−0.358, *p* < 0.001) and ‘friends’ support (−0.279, *p* < 0.001).’ These results align with research conducted in other East Asian contexts. As an example of this, a recent study in Korea found that greater satisfaction with family life was linked to a reduction in suicidal thoughts and emphasized that strategies promoting open communication within the family can strengthen relationships and improve psychological well-being (Jeong-mi & Ji-hoon, 2024).

The interactions in the regression model only including the predictors MSPSS and ICQ-15 with the dependent variables C-SSRS and PHQ-9 showed how interpersonal competence and perceived social support are negatively associated with depression and suicidal risk, fulfilling H1, although in a different way in the proportion of significance. In the Spanish sample, correlations between both protective constructs (MSPSS and ICQ-15) and PHQ-9 or C-SSRS were mostly non-significant, except for a small negative association between support from a ‘significant other’ and suicidal risk. In contrast, in the Japanese sample, several significant negative associations emerged; self-disclosure correlated with lower depressive symptoms, while the ability to initiate relationships and manage conflicts was linked to lower suicide risk. In the study of Ishiguro (2023), it was found that brief interactions were positively associated with happiness, mediated by a reduction in loneliness. This reflects how in Japan, the ability to initiate, maintain, and manage interactions can mitigate the perception of interpersonal disconnection and modify its relationship with suicide risk. Furthermore, family support was the main protective factor against both depression and suicidal risk, followed by support from significant others and friends. These findings suggest that interpersonal competence and perceived social support may take their effect more indirectly or might depend on other psychosocial conditions in the Spanish sample. Internal resources, such as a sense of coherence or self-esteem when communicating (da-Silva-Domingues et al., 2025), may enable individuals to fully benefit from social support and enhance its protective effect against depression or suicidal ideation.

A current study on mental well-being in Spain found perceived loneliness to be a strong predictor of mental health outcomes and found that social support partially mediates the relationship between loneliness and overall well-being (Egaña-Marcos et al., 2025). Together, these findings highlight the complex interaction between risk and protective factors in shaping mental health outcomes. The H2 was not completely empirically confirmed. Together with the independent variables in the complex regression models, the interactions between the moderators were tested, and the three-way and four-way interactions did not reach statistical significance in the different models. However, the theoretical model and the direction of the coefficients suggest a pattern consistent with the hypothesis: greater support or interpersonal competence are associated with lower risk. The interaction coefficient of INQ-PB and INQ-TB increased its value from 0.0004 to 0.0019 when adding MSPSS to the model explaining suicidal ideation (C-SSRS).

The study of Aizpurua et al. (2021), conducted among university students in Spain, found that higher levels of social support were associated with a reduced risk of suicidal ideation and behavior, identifying socialization and resilience as protective factors. Additionally, another study with a psychiatric population proved that INQ-TB and INQ-PB in combination were proximal risk factors for suicidal ideation when the patients had lower perceived social support (Sparks et al., 2023), so the protection role of it was expected. In contrast to this, both in the SEM model and in the logistic regression, coefficients associated with suicidal behavior adding ICQ-15 to ideation produced negative values (−0.001 and −1.056 respectively), indicating inverse relationships, regardless of significance, and positive values when also adding ACSS (0.000 and 0.026). These results altogether answer questions about the existence of differences in the relative association of thwarted belongingness and perceived burdensomeness with suicidal ideation, when adjusted for social support, as well as the moderating role of interpersonal competence between acquired capability for suicide and the transition from ideation to suicidal behavior.

Contrary to expectations, interpersonal competence (ICQ-15) did not have a direct effect on the transition to suicidal behavior. This could suggest that, in young adults, other interpersonal factors such as perceived burden or social support play a more relevant role in the ITS variables. These findings echo the notion from close relationship research that not all interpersonal variables, such as global relational satisfaction or general communication, exert a direct effect on suicidal ideation; rather, more specific relational dynamics, like perceived burdensomeness or culturally relevant social support, may play a more decisive role (Love & Morgan, 2024).

Social support and interpersonal competence may have a differential impact in each cultural context, as the coefficient interaction between MSPSS with INQ-TB indicated a negative tendency in the Japanese sample compared to the Spanish sample in explaining suicidal ideation, and the coefficient was significant for depression when ICQ-15 was added to INQ-TB in the Japanese (1.166 × 10^−1^, *p* < 0.05). Although this positive association in the Japanese may seem counterintuitive, it aligns with prior research showing that even being competent in communicating with others may benefit people when others’ reactions are characterized by listening, empathy, or support. This suggests that in Japan, the benefits of disclosure are contingent on the social context and the perceived quality of interpersonal responses (Taku et al., 2009). Regarding H3, the results partially confirm that the association between perceived burdensomeness (INQ-PB), thwarted belongingness (INQ-TB), and suicidal ideation differ in magnitude across cultures. Specifically, the effect of INQ-PB on suicidal ideation is significantly weaker in the Japanese sample compared to the Spanish sample, as indicated by the interaction term between INQ-PB and country (−0.118, *p* < 0.01). This may be attenuated by more permissive attitudes toward suicide in Japanese society and limited engagement in suicide prevention (Otsuka et al., 2020).

Similarly, the effect of INQ-TB is also lower in Japan (−0.042, *p* < 0.05).

The main effect of country further indicated lower suicidal ideation in Japan overall (−0.990, *p* < 0.001). These results suggest that the interpersonal constructs proposed by the ITS operate differently depending on cultural values related to emotional expression and relational interdependence. In collectivist contexts such as Japan, feeling like a burden for close relatives or experiencing low belongingness might be perceived as less personally threatening or more socially normative than in individualist societies like Spain, where autonomy and self-sufficiency are more strongly emphasized. A cross-cultural study on hikikomori profiles showed that social avoidance and lack of emotional support were positively associated with thwarted belongingness and perceived burdensomeness and that the relationship between social avoidance and perceived burdensomeness was stronger in Japanese participants (Taku et al., 2023). However, in individualistic societies such as Spain where autonomy and self-reliance are valued, the same feelings of burdensomeness and low belongingness might be perceived as more problematic and associated with a higher risk of suicidal ideation.

Finally, H4, which refers to the role of acquired capability for suicide in moderating the transition from suicidal ideation to behavior, was partially supported, without clear evidence of moderation by culture. The effect of this risk variable was significant and positive on suicidal behavior in SEM (*p* = 0.029) but only positive in the logistic regression. Although no clear evidence was found about culture in moderating this effect, what is evident is that becoming used to self-harming increases the risk of suicidal behavior (Fernández-Montalvo et al., 2021).

Focusing on the PHQ-9 results, we can answer questions about the similarity or difference between interpersonal and cultural factors that explains suicide risk and depressive symptoms. After executing regression analysis with the moderator variables ‘interpersonal competence’ and ‘perceived social support’, they were negatively related with ‘depression’, although the relationship with interpersonal competence was not significant (B = –0.170, *p* = 0.690). Regarding ‘suicide risk’, only ‘perceived social support’ was significantly negatively related; with ‘interpersonal competence’, the coefficient value was slightly positive and non-significant (0.078, *p* = 0.553). These findings align with previous evidence from the Japanese working population, where support from co-workers and family was associated with lower depressive symptoms (co-workers: *p* = 0.016; family: *p* = 0.001) (Omichi et al., 2022). Comparing cultural contexts, the regression models suggested small cultural differences in PHQ-9 results but significant differences in ‘suicide risk’, as the Spanish sample had low scores compared with the Japanese. When applying model 2 in the PHQ-9 with social support as a moderator of depression, we found that in the Japanese sample, social support was less protective against the effect of thwarted belongingness on depression; in Spain, however, the protection was clearer. These results align with recent Spanish longitudinal research (Erdem et al., 2025) on social pressure in digital contexts, underscoring the crucial role of social support in mitigating depressive symptoms.

The interaction coefficient between INQ-PB and INQ-TB decreased in value when adding MSPSS from 0.0003 to 0.0002, but only when explaining depression (PHQ-9). Contrary to suicide risk results, in the Japanese sample, when adding MSPSS to INQ-TB, the coefficient was positive and significant (0.08, *p* < 0.001). With INQ-PB, there was a negative but not significant coefficient. The ICQ-15 stood out significantly for Japan when added to INQ-TB (1.166 × 10^−1^, *p* < 0.05) and showed a difference between a positive coefficient and a negative one; this was not significant when adding it to the ITS variables (INQ-PB, INQ-TB and ACSS) in the Japanese sample (−1.584 × 10^−4^, *p* = 0.84). This is consistent with a recent study (Ishiguro, 2023) showing that even very brief forms of social interaction—or minimal social interactions—can improve subjective well-being through their impact on the sense of belonging. This suggests that in cultural contexts where social norms tend to inhibit self-disclosure or the explicit seeking of support, interpersonal skills and everyday micro-interactions acquire a particularly marked protective value, directly influencing the sense of belonging. Sex differences were only highlighted in the PHQ-9 results, as they became irrelevant in the suicide risk analyses. Sex was significantly associated with depression, with men showing lower depressive symptoms compared to women. These findings are consistent with both Spanish and Japanese studies that report higher incidence in women during youth (Knowles et al., 2025; Ozamiz-Etxebarria et al., 2023; Sugawara et al., 2015). 

### 4.2. Limitations and Strengths

The main limitations of this study are due to the characteristics of the sample. As noted in our previous study using the same sample, the limited size and the restricted age range (18–40 years) force us to be cautious in generalizing the findings. Although the sample used was non-clinical, which may limit the representativeness of suicidal behavior, the study provides insight into the mechanisms of the ITS in community populations, which is also necessary for prevention interventions. Another bias that arises from the way the data was collected (self-reported measures) is social desirability. Nevertheless, this approach is standard in community-based suicide research.

Due to the binary nature of suicidal behavior and the inclusion of multiple interactions, the SEM model was saturated, and logistic regression was, consequently, applied as a more appropriate method, providing robust and interpretable estimates in spite of the different outcomes. Instead of using a latent variable (suicidal ideation derived from perceived burdensomeness and thwarted belongingness), suicidal ideation scores were used directly from the C-SSRS, which simplifies the interpretation of direct and interactive effects, although it does not account for measurement error.

Notwithstanding the facts aforementioned, this study presents several key strengths, as it is grounded in the ITS (Joiner, 2005), providing a solid theoretical framework to examine mechanisms underlying suicidal ideation and behavior. The interactions with the moderators (interpersonal competence and social support) are not strictly part of the core model of the ITS, but they are consistent with subsequent empirical extensions and developments, which explore which factors buffer or aggravate the perception of burdensomeness and frustrated belonging. Otherwise, the cross-cultural design, including samples from Spain and Japan, allows for the exploration of cultural similarities and differences in risk and protective factors. Finally, the use of different robust analytical methods, including SEM and logistic regression, enabled the examination of complex relationships and interactions between variables.

### 4.3. Future Research

Even though the cross-sectional design of this study does not allow for confirmatory causal inferences, it provides inferences of interpersonal and cultural factors influencing suicide risk in young adults. Future research should build on these findings by conducting longitudinal studies to examine causal pathways and the temporal dynamics of suicidal ideation and behavior. Exploring the conditions under which interpersonal competence and perceived social support exert a protective effect on suicidal risk could clarify whether interpersonal competence and social support act indirectly, enhancing connectedness and reducing feelings of burdensomeness or thwarted belongingness. Additionally, replication in clinical samples, where suicidal behavior is more frequent and variable, would help to validate and extend the current results, providing insights relevant for both prevention and intervention strategies.

### 4.4. Implications for Clinical and Academic Practice

We should take the following steps in the aftermath of this research:

- Integrate assessments of key interpersonal factors in suicidal behavior—such as thwarted belongingness, perceived burdensomeness, and acquired capability for suicide—into routine clinical screening protocols, given their relevance across cultural contexts.
- Prioritize interventions that strengthen interpersonal functioning through psychoeducational, community-based, and relational skill-building programs, particularly in settings where social disconnection is a key risk factor.
- Develop culturally responsive prevention and intervention strategies, acknowledging cross-cultural differences in the expression of interpersonal risk and in patterns of help-seeking.
- Enhance professional training so clinicians understand how communicative and relational processes interact with suicidal vulnerability, enabling earlier and more informed intervention.
- Promote research lines that incorporate interpersonal competence and social support into explanatory models of suicidal behavior, integrating dispositional, interpersonal, and contextual variables.

## 5. Conclusions

The present study empirically tested relationships between suicide risk variables proposed by the ITS, allowing for a more precise examination of the mechanisms linking interpersonal factors to suicidal behavior, unlike our previous analyses. By incorporating interpersonal competence and perceived social support as moderating variables, this study expands the interpersonal framework, highlighting the potential protective role of these factors in the pathway leading to the formation of suicidal ideation and its transition to suicidal behavior. Although interpersonal competence showed weaker associations than expected with suicidal behavior, the weight of the core mechanisms (perceived burdensomeness and thwarted belongingness) suggested that maybe it is not the ability to communicate or the act of talking to others that truly protects against suicide, but rather how the person feels in relation to others in those interpersonal processes (valued or connected).

Furthermore, comparison with a dependent variable such as depression allows us to examine whether those interpersonal factors exert similar or distinct influences on each outcome, as social support clearly had a protective effect against depression, whereas for suicidal risk, its effects were more limited. Cross-cultural comparisons contribute to a culturally sensitive understanding of the different interpersonal mechanisms underlying protective factors. Empathic responses to self-disclosure enhance protective effects, especially in collectivist societies, highlighting that the quality of interactions and subjective social connection, rather than the mere presence of support, are key psychological mechanisms for both suicide risk and depression.

Finally, the results underscore the importance of promoting interpersonal and communicative competencies in suicide prevention programs, enhancing the quality of interpersonal responses from the individual’s close relational environment to maximize their protective effects, particularly among young adults, whose developmental stage involves the consolidation of purpose and sense of belonging (dimensions that are still malleable and thus responsive to preventive and educational interventions).

## Figures and Tables

**Table 1:** Percentages of the new sociodemographic variables in the total sample.

	Percentages
Mental health	
With diagnosis	69.3%
Without diagnosis	20.6%
Minor problems or prefer not to answer	10.1%
Physical health	
With diagnosis	74.4%
Without diagnosis	23.3%
Minor problems or prefer not to answer	2.3%
Receiving or having received any type of treatment in the last 6 months?	
None	72.4%
Psychological treatment	9.8%
Pharmacological treatment	5.5%
Psychological and pharmacological treatment	2.5%
Without specifying	9.6%
Self-help material	0.2%
Economic status	
High class	11%
Middle class	45.5%
Low class	43.5%
COVID-19 repercussions ^1^	
Relationships	33.4%
Mental health	29.5%
Physical health	25.2%
Stress or home workload	16.7%
Work	7.3%
Studies	3.9%
Place of residence	3.2%
Without disturbances	28.6%
Perceived an improvement	11.2%

^1^ The percentages do not add up to 100%, as multiple answers were allowed.

**Table 2:** Predictors of depression (PHQ-9): hierarchical regression results including MSPSS.

Coefficients	Estimate	Std. Error	t Value	Pr(>|t|)
(Intercept)	13.9214673	0.3555541	39.154	<2 × 10^−16^ ***
INQ-PB ^1^	1.2417304	0.0592886	20.944	<2 × 10^−16^ ***
INQ-TB ^2^	0.0222845	0.0225941	0.986	0.324556
MSPSS ^3^	−0.0400484	0.2242487	−0.179	0.858346
Country (Japan)	−4.4805398	0.4851050	−9.236	<2 × 10^−16^ ***
Sex (men)	−0.9315542	0.3173418	−2.935	0.003514 **
INQ-PB ^1^: country (Japan)	−0.9161424	0.0692362	−13.232	<2 × 10^−16^ ***
INQ-PB ^1^–INQ-TB ^2^–MSPSS ^3^	0.0002075	0.0024478	0.085	0.932482
INQ-TB ^2^–MSPSS ^3^–country (Japan)	0.0874152	0.0261123	3.348	0.000889 ***
INQ-PB ^1^–INQ-TB ^2^–MSPSS ^3^–country (Japan)	0.0015839	0.0028892	0.548	0.583847

Signif. codes: 0 ‘***’ 0.001 ‘**’. ^1^ INQ-PB: measure of perceived burdensomeness. ^2^ INQ-TB: measure of thwarted belongingness. ^3^ MSPSS: measure of perceived social support.

**Table 3:** Three-way interactions on PHQ-9 results including MSPSS.

	Coef.	z-Value	t Value	*p*-Value
INQ-PB ^1^: INQ-TB ^2^–MSPSS ^3^	0.0002075	0.0024478	0.085	0.932482
INQ-PB ^1^–INQ-TB ^2^–country	0.0072468	0.0054916	1.320	0.187684
INQ-PB ^1^–MSPSS ^3^–country	−0.0341520	0.0472690	−0.723	0.470388
INQ-TB ^2^–MSPSS ^3^–country	0.0874152	0.0261123	3.348	0.000889 ***

Signif. codes: 0 ‘***’. ^1^ INQ-PB: measure of perceived burdensomeness. ^2^ INQ-TB: measure of thwarted belongingness. ^3^ MSPSS: measure of perceived social support.

**Table 4:** Two-way interactions on PHQ-9 results including MSPSS.

	Coef.	z-Value	T Value	*p*-Value
INQ-PB ^1^–INQ-TB ^2^	0.0003624	0.0037519	−0.052	0.958512
INQ-TB ^2^–MSPSS ^3^	−0.0018153	0.0149069	−0.122	0.903133
INQ-PB ^1^–MSPSS ^3^	−0.0019168	0.0368250	0.420	0.675204
INQ-TB ^2^–country(Japan)	0.0467895	0.0460121	1.017	0.309790
INQ-PB ^1^–country(Japan)	−0.9161424	0.0692362	−13.232	<2 × 10^−16^ ***
MSPSS ^3^–country(Japan)	−0.5778509	0.3816238	−1.514	0.130732

Signif. codes: 0 ‘***’. ^1^ INQ-PB: measure of perceived burdensomeness. ^2^ INQ-TB: measure of thwarted belongingness. ^3^ MSPSS: measure of perceived social support.

**Table 5:** Predictors of depression (PHQ-9): hierarchical regression results including ICQ-15.

Coefficients	Estimate	Std. Error	t Value	Pr(>|t|)
(Intercept)	1.384 × 10^1^	3.553 × 10^−1^	38.963	<2 × 10^−16^ ***
INQ-PB ^1^	1.230 × 10^0^	6.080 × 10^−2^	20.230	<2 × 10^−16^ ***
INQ-TB ^2^	2.346 × 10^−2^	2.399 × 10^−2^	0.978	0.32865
ACSS ^3^	4.899 × 10^−2^	5.083 × 10^−2^	0.964	0.33577
ICQ-15 ^4^	3.392 × 10^−1^	4.730 × 10^−1^	0.717	0.47362
Country (Japan)	−4.390 × 10^0^	4.882 × 10^−1^	−8.992	<2 × 10^−16^ ***
Sex (men)	−9.573 × 10^−1^	3.361 × 10^−1^	−2.848	0.00463 **
INQ-PB ^1^–country (Japan)	−8.990 × 10^−1^	7.111 × 10^−2^	−12.643	<2 × 10^−16^ ***
INQ-TB ^2^–country (Japan)	8.272 × 10^−2^	4.281 × 10^−2^	1.933	0.05399 .
INQ-TB ^2^–ICQ-15 ^4^–country (Japan)	1.166 × 10^−1^	4.785 × 10^−2^	2.436	0.01529 *
INQ-PB ^1^–INQ-TB ^2^–ACSS ^3^–country (Japan)	1.987 × 10^−3^	7.639 × 10^−4^	2.601	0.00963 **
INQ-PB ^1^–ACSS ^3^–ICQ-15 ^4^–country (Japan)	2.589 × 10^−2^	1.494 × 10^−2^	1.733	0.08378 .
INQ-PB ^1^–INQ-TB ^2^–ACSS ^3^–ICQ-15 ^4^–country (Japan)	−1.584 × 10^−4^	8.007 × 10^−4^	−0.198	0.84332

Signif. codes: 0 ‘***’ 0.001 ‘**’ 0.01 ‘*’ 0.05 ‘.’. ^1^ INQ-PB: measure of perceived burdensomeness. ^2^ INQ-TB: measure of thwarted belongingness. ^3^ ACSS: measure of acquired capability for suicide. ^4^ ICQ-15: measure of interpersonal competence.

**Table 6:** Main ordinal regression results on C-SSRS including MSPSS.

Coefficients	Estimate	Std. Error	t Value	95% CI
INQ-PB ^1^	0.2124805	0.037311	5.69484 ***	[0.14, 0.29]
INQ-TB ^2^	0.0399497	0.013608	2.93578 **	[0.01, 0.07]
INQ-PB ^1^–INQ-TB ^2^	0.0004415	0.002473	0.17854	[−0.01, 0.01]
MSPSS ^3^	−0.2410380	0.134165	1.79658	[−0.51, 0.02]
Country (Japan)	−0.9895160	0.288263	−3.43269 ***	[−1.56, −0.43]
INQ-PB ^1^–country (Japan)	−0.1183357	0.042067	−2.81305 **	[−0.20, −0.04]
INQ-TB ^2^–country (Japan)	0.0671981	0.027125	−2.47737 *	[−0.12, −0.01]
INQ-PB ^1^–INQ-TB ^2^–MSPSS ^3^	0.0019709	0.001640	1.20188	[−0.00, 0.01]
INQ-TB–MSPSS ^3^–country (Japan)	−0.0290619	0.015644	−1.85775	[−0.06, 0.00]
INQ-PB ^1^–INQ-TB ^2^–MSPSS ^3^–country (Japan)	−0.0008124	0.001925	−0.42198	[−0.01, 0.00]

* |t| > 1.96 = *p* < 0.05. ** |t| > 2.58 = *p* < 0.01. *** |t| > 3.29 = *p* < 0.001. ^1^ INQ-PB: measure of perceived burdensomeness. ^2^ INQ-TB: measure of thwarted belongingness. ^3^ MSPSS: measure of perceived social support.

**Table 7:** SEM results on suicidal behavior, adding the predictor ACSS and the moderator ICQ-15.

Coefficients	Estimate	Std. Error	z-Value	P(>|z|)	Std.lv	Std.all
Predicted ideation	−0.001	0.001	−0.525	0.599	−0.001	−0.074
ACSS ^1^	0.023	0.014	1.641	0.101	0.023	0.149
ICQ-15 ^2^	−0.138	0.112	−1.230	0.219	−0.138	−0.101
Predicted ideation–ACSS ^1^	0.000	0.000	0.777	0.437	0.000	0.104
Predicted ideation–ICQ-15 ^2^	−0.001	0.002	−0.651	0.515	−0.001	−0.136
Predicted ideation–ACSS ^1^–ICQ-15 ^2^	0.000	0.000	0.219	0.826	0.000	0.047

^1^ ACSS: measure of acquired capability for suicide. ^2^ ICQ-15: measure of interpersonal competence.

**Table 8:** Logistic regression coefficients for suicidal behavior.

Coefficients	Estimate	z-Value	P(>|z|)	OR	95% CI
Suicidal Ideation	2.618	3.351	0.0008 **	13.7	3.98–103.27
ACSS ^1^	−0.040	−0.418	0.676	0.96	0.78–1.15
ICQ-15 ^2^	0.750	0.795	0.426	2.12	0.41–17.92
Ideation–ACSS ^1^	0.105	1.066	0.286	1.11	0.92–1.37
Ideation–ICQ-15 ^2^	−1.056	−1.087	0.277	0.35	0.04–1.92
Ideation–ACSS ^1^–ICQ-15 ^2^	0.026	0.933	0.351	1.03	0.97–1.09
Sex (women)	0.090	0.282	0.778	1.09	0.59–2.05
Country (Japan)	1.256	3.726	0.0002 **	3.51	1.84–6.96

** *p* < 0.001. ^1^ ACSS: measure of acquired capability for suicide. ^2^ ICQ-15: measure of interpersonal competence.

## Data Availability

Research data are not shared as part of an ongoing study. The statistical data, as well as the sociodemographic data obtained through the survey administered to the participants, remain in the custody of the authors for reasons of confidentiality, unless externally requested from the corresponding author; they may be delivered only upon encoding all pertinent socio-demographic data in order to preserve the patients’ identity. The original contributions presented in the study are included in the article; further inquiries can be directed to the corresponding author. All data relating to this research, which is part of the doctoral thesis of the main researcher of this study, will be part of the repository of the University of Murcia, and this institution undertakes responsibility for safeguarding them for the period stipulated.

## Supplementary Materials

The following supporting information can be downloaded at: https://www.mdpi.com/article/10.3390/ejihpe16010004/s1, Supplementary Material S1. Preliminary analyses. Supplementary Material S2. Descriptive results of the MSPSS and ICQ-15. Supplementary Material S3. Direct effects of social support and interpersonal competence on suicide risk and depression. Supplementary Material S4. P-P plot of the residuals for PHQ-9. Supplementary Material S5. Simple slopes plot for the interaction between INQ-TB and MSPSS. Supplementary Material S6. Country-specific interaction effects of ITS variables.
