# Supplementary material for: Interpersonal Determinants of Suicide Risk Among Young Adults: A Cross-Cultural Study
Source: Eur J Investig Health Psychol Educ. 2025 Dec 24;16(1):4. doi: 10.3390/ejihpe16010004 (PMC12840238; doi:10.3390/ejihpe16010004)
Supplement: Supplementary file 1 [file ejihpe-16-00004-s001.zip › Supplementary Material S6.pdf]

**Supplementary Material S6.** Country-specific interaction effects of ITS variables.

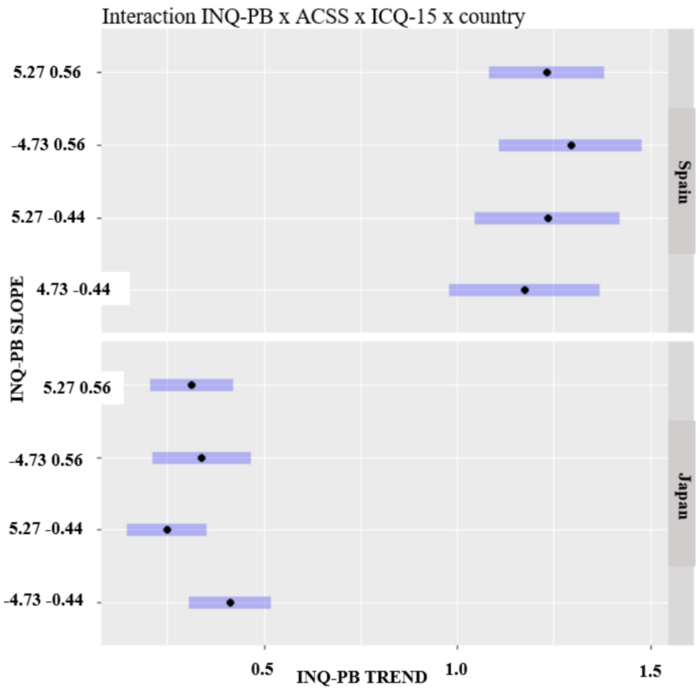


**Figure S3.** Interaction INQ-PB x ACSS x ICQ-15 x country.

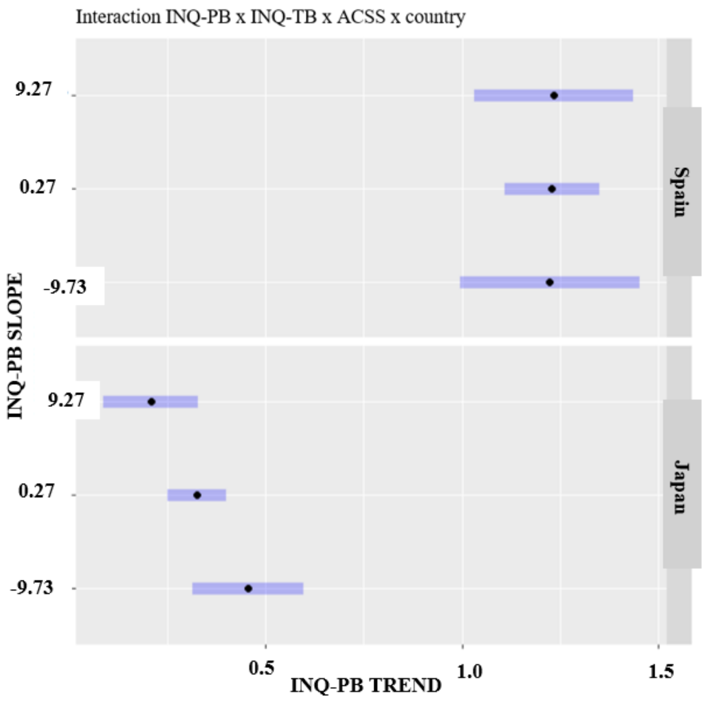


**Figure S4.** Interaction INQ-PB x INQ-TB x ACSS x country.
