# Supplementary material for: Interpersonal Determinants of Suicide Risk Among Young Adults: A Cross-Cultural Study
Source: Eur J Investig Health Psychol Educ. 2025 Dec 24;16(1):4. doi: 10.3390/ejihpe16010004 (PMC12840238; doi:10.3390/ejihpe16010004)
Supplement: Supplementary file 1 [file ejihpe-16-00004-s001.zip › Supplementary Material S1.pdf]

Supplementary Material S1. Preliminary analyses

Firstly, the relevant analyses were carried out to verify the adequate psychometric properties of the C-SSRS screen version (Posner et al., 2011) in its Spanish and Japanese versions. Starting with a CFA the model remained as 3 factors: ideation = item 1 + item 2; planning = item 3 + item 6.1; and intention/action = item 4 + item 5 + item 6.2. CFI = 0.999 and TLI = 0.999 values showed excellent fit compared to the independent model, and a RMSEA = 0.086 resulted moderately acceptable. Although the SRMR = 0.100 was above the ideal threshold, it does not indicate that the model is completely inadequate. Regarding the standardized factor loadings in both groups (Spanish and Japanese), in Table S2 and Table S3 we can see the different values, all enough high and significant. Some of them slightly exceed 1 due to the small number of items per factor and high correlations between them, but the interpretations of the model relationships were consistent with the theory. Regarding covariances between factors, in both countries were high, while in the Japanese sample the values were between 0.727-1.262, in the Spanish they were between 0.846–1.095. Using the C-SSRS screen version for both suicidal ideation and behavior, we separated the items of this measure between 2 scales (ideation and intention/action) in order to check the influence of capability for suicide in the process to suicidal ideation to action.

Table S2. Standardized factor loadings in the Japanese C-SSRS screen-version.

	Estimate	P (>|z|)	Std.lv	Std.all
Ideation				
item 1	1.000		0.658	0.658
item 2	1.831	0.000	1.204	1.204
Ideation with planning				
item 3	1.000		0.774	0.774
item 6.1	0.829	0.000	0.642	0.642
Intention/ action				
item 4	1.000		1.011	1.011
item 5	0.944	0.000	0.955	0.955
item 6.2	0.756	0.000	0.765	0.765

Table S3. Standardized factor loadings in the Spanish C-SSRS screen-version.

	Estimate	P (>|z|)	Std.lv	Std.all
Ideation				
item 1	1.000		0.954	0.954
item 2	1.055	0.000	1.007	1.007
Ideation with planning				
item 3	1.000		0.927	0.927
item 6.1	0.942	0.000	0.873	0.873
Intention/ action				
item 4	1.000		0.995	0.995
item 5	1.003	0.000	0.998	0.998

item 6.2	0.807	0.000	0.803	0.803

According to the model that differentiates ideation from behavior: items 1-5 were grouped in 'ideation' with a value of Cronbach's alpha = 0.86, indicating a very good internal consistency. The 95% CI: 0.84–0.88 suggested that this subscale is consistent and reliable, the std. alpha estimated homogeneity of the items with a value of 0.87 and the average correlations between them (0.58) was adequate, as any of the items significantly reduced consistency if removed. Through the Omega hierarchical analysis, α got a similar value (0.87), and the reliability estimated for a general factor (g) (0.90), total omega (0.95) and hierarchical omega (0.78), suggested that g explained most of the shared variance. Analyzing the factorial structure, g explained 65% of the common variance. RMSEA= 0.422 was considered very high, also the inclusion of group factors added to explain residual variance and the correlations with the factors (≥0.88) indicated the scores were reliable and representative of the factor. Regarding items 6.1 and 6.2, as there were only 2 items, their means, deviations and frequencies were obtained. For item 6.1: mean= .13, D= .332 and the frequency marked 'yes' was 55 (12.6%); and for item 6.2: mean= .03, D= .182 and frequency marked 'yes' 15 (3.4%). The correlation between these 2 items was r= .497 (> 0.3-0.4), indicating consistency enough to consider them a measure for suicidal behavior.
