# Supplementary material for: Interpersonal Determinants of Suicide Risk Among Young Adults: A Cross-Cultural Study
Source: Eur J Investig Health Psychol Educ. 2025 Dec 24;16(1):4. doi: 10.3390/ejihpe16010004 (PMC12840238; doi:10.3390/ejihpe16010004)
Supplement: Supplementary file 1 [file ejihpe-16-00004-s001.zip › Supplementary Material S2.pdf]

Supplementary material S2. Descriptive results of the MSPSS and ICQ-15

Following up with the scores on the MSPSS and ICQ-15 in the Spanish and Japanese samples, the results are presented below showing the trends of each of the samples. As we can see in Table S4 and Table S5:

Table S4. MSPSS scores in both samples.

			Spain				Japan		d Cohen's
MSPSS areas	Mean	SD	Skew.	Kurt.	Mean	SD	Skew.	Kurt.	
Significant others	5.60	1.74	–1.24	.40	5.18	1.73	-.79	-.49	0.26(small)
Family	5.02	1.80	-.62	-.85	4.78	1.73	-.58	-.79	0.24(small)
Friends	5.25	1.82	-.95	-.14	4.72	1.73	-.53	-.78	0.13(neglig.)
Total	5.29	1.49	-.91	.06	4.90	1.52	-.70	-.35	0.30(small)

Table S5. ICQ-15 scores in both samples.

			Spain				Japan		d Cohen's
ICQ-15 subscales	Mean	SD	Skew.	Kurt.	Mean	SD	Skew.	Kurt.	
Initiation of a relationship	3.04	1.14	-.08	-.95	3.03	.95	-.24	-.35	0.33(small)
Negative assertion	3.18	1.10	-.17	-.76	3.46	.87	-.56	-.27	-0.41(small)
Emotional support assertion	4.32	.75	-1.57	3.25	3.49	.86	-.71	-.41	-0.04(neglig.)
Disclosure	3.20	1.15	-.24	-.91	3.26	.92	-.26	-.23	-0.10 (neglig.)
Conflict management	3.9	.75	-.76	.62	3.21	.89	-.25	-.32	1.41(large)
Total	3.54	.73	-.39	.11	3.29	.77	-.51	.55	0.84(large)

We probed with Levene's Test the equality of variances for the MSPSS and ICQ-15, and for both equal variances were assumed ($F = 0.026$; $p = 0.872 \rightarrow p > 0.05$ and $F = 0.020$; $p = 0.889 \rightarrow p > 0.05$). Using t-test for equality of means, we got a highly significant difference in the ICQ-15 scores ($t = 3.397$; $df = 435$; $p = 0.001$) and a significant difference in the MSPSS ($t = 2.682$; $df = 435$; $p = 0.008$). The Spanish sample had a higher mean than Japanese (Difference in Means = 0.24958, Mean difference = 0.39297 with MSPSS).
