# Supplementary material for: Interpersonal Determinants of Suicide Risk Among Young Adults: A Cross-Cultural Study
Source: Eur J Investig Health Psychol Educ. 2025 Dec 24;16(1):4. doi: 10.3390/ejihpe16010004 (PMC12840238; doi:10.3390/ejihpe16010004)
Supplement: Supplementary file 1 [file ejihpe-16-00004-s001.zip › Supplementary Material S3.pdf]

**Supplementary material S3.** Direct effects of social support and interpersonal competence on suicide risk and depression

As previously reported (Martínez-Rives et al., 2025d), the dependent and independent variables showed correlations with acceptable values. For example, PHQ-9 scores showed strong positive correlations with INQ-PB (e.g., $r = .62$ in the Japanese sample; $r = .97$ in the Spanish sample) and moderate correlations with INQ-TB ($r \approx .48$–$.59$), whereas associations with ACSS-FAD were weak. Simple correlations were performed to analyze which areas of social support, and which dimensions of competence have more weight on the PHQ-9 and C-SSRS results:

In both samples, all Pearson and Spearman correlation analyses performed showed that all areas of MSPSS were strongly correlated with each other (coefficients ranging from .492 to .723, $p < 0.01$), suggesting that those who report higher support in one area also tend to report higher support in the other areas, and between all dimensions of the ICQ-15 (coefficients ranging from .355 to .728, $p < 0.01$), alluding a relation between the interpersonal skills. In the Spanish sample, any area of the MSPSS correlated significantly with PHQ-9 results.

The Spearman correlations between MSPSS and C-SSRS showed a small significant correlation between support received from a 'significant other' ($\varrho = -0.124$, $p = 0.045$). Spearman correlations between the dimensions of the ICQ-15 and C-SSRS were not significant, and only one of them was negative, 'disclosure' (-.010, $p = .871$). Although most Pearson correlations between the dimensions of the ICQ-15 and PHQ-9 were negative, none were significant.

In the Japanese sample, simple Pearson correlations between ICQ-15 with PHQ-9 results showed a significant correlation with the 'disclosure' dimension ($r = -0.155$, $p = 0.039$). Although the other dimensions showed negative trends, they were not significant. Regarding Spearman correlations between ICQ-15 with C-SSRS, they revealed different important dimensions, as significant negative correlations were found with 'initiating relationships' ($\varrho = -0.206$, $p = 0.006$) and 'conflict management' ($\varrho = -0.198$, $p = 0.008$). Spearman correlations were also analyzed between MSPSS areas and C-SSRS, where only the negative correlation with 'family' support ($\varrho = -0.289$, $p < 0.01$) turned out significant. Different results were found between the MSPSS with PHQ-9, with 'family' support being the most associated (-.408, $p < .001$), followed by 'significant others' support (-.358, $p < .001$) and 'friends' support (-.279, $p < .001$).'
