# Supplementary material for: Interpersonal Determinants of Suicide Risk Among Young Adults: A Cross-Cultural Study
Source: Eur J Investig Health Psychol Educ. 2025 Dec 24;16(1):4. doi: 10.3390/ejihpe16010004 (PMC12840238; doi:10.3390/ejihpe16010004)
Supplement: Supplementary file 1 [file ejihpe-16-00004-s001.zip › Supplementary Material S5.pdf]

Supplementary material S5. Simple slopes plot for the interaction between INQ-TB and MSPSS.

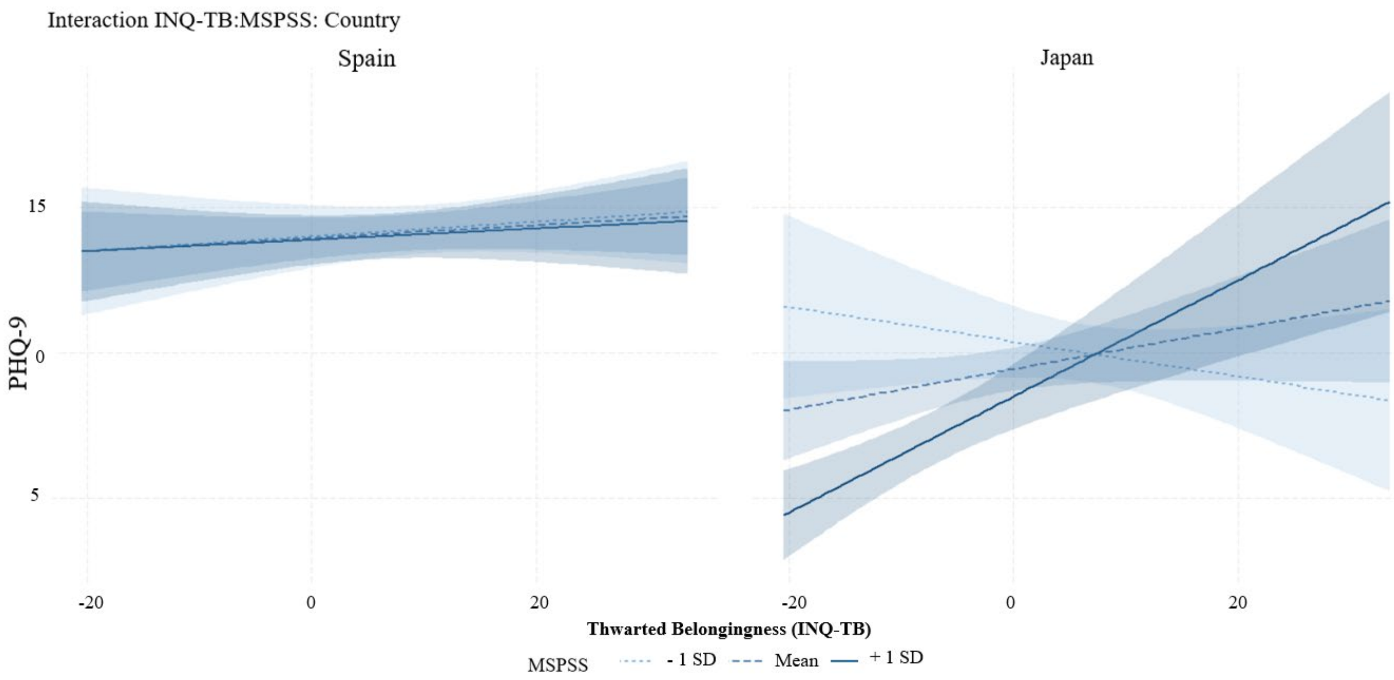

Figure S2. Simple slopes plot for the interaction between INQ-TB and MSPSS. X-axis represents INQ-TB scores; Y-axis indicates PHQ-9 scores.
